# Predicting Serious Adverse Events, Medication Abuse, Misuse, and Risk of Dependence for Medications with High Dependence Potential: Role of Patient-Reported Factors and Machine Learning Approach

**DOI:** 10.3390/healthcare14101265

**Published:** 2026-05-07

**Authors:** Yujin Kim, Yu Jin Sohn, Jin Young Yoo, Minsung Kim, Semi Kim, Yeo Jin Choi

**Affiliations:** 1 Department of Regulatory Science, Graduate School, Kyung Hee University, Seoul 02447, Republic of Korea; 2 Institute of Regulatory Innovation Through Science (IRIS), Kyung Hee University, Seoul 02447, Republic of Korea; 3 College of Pharmacy and Institute of Integrated Pharmaceutical Sciences, Kyung Hee University, Seoul 02447, Republic of Korea

**Keywords:** pharmacovigilance, machine learning, opioids, signal detection, drug abuse, drug dependence, drug misuse

## Abstract

**Background/Objectives**: This study aimed to evaluate the frequency and predictors of adverse drug events (ADEs) related to medication abuse, misuse, and dependence, along with serious adverse events (SAEs), and to develop machine learning models to detect serious abuse, misuse, and dependence cases. **Methods**: This study included 455,415 ADE reports involving medications with high dependence potential reported to the Korea Adverse Event Reporting System (KIDS KAERS DB) between 2013 and 2022. Multivariate logistic regression was used to identify predictors. Three machine learning algorithms, random forest (RF), support vector machine, and eXtreme Gradient Boosting, were developed and evaluated. **Results**: Higher reporting likelihood of abuse-, misuse-, and dependence-related ADEs was observed with concomitant use of acetaminophen (OR 3.60, 95% CI 2.40–5.39), antidepressants (OR 1.75, 95% CI 1.17–2.61), antipsychotics (OR 4.97, 95% CI 3.21–7.17), and anticonvulsants (OR 3.42, 95% CI 2.42–4.81). Reports from the general public were associated with higher odds of abuse, misuse, and dependence than those from healthcare professionals (OR 4.59, 95% CI 3.04–6.94). Ketamine (ROR 14.03) and bromazepam (ROR 13.02) showed the highest likelihood of being classified as SAEs. Cardiovascular (ROR 30.36) and respiratory disorders (ROR 17.03) demonstrated the highest SAE reporting likelihood. RF model demonstrated the best predictive performance (AUC-ROC 0.928; accuracy 94.4%), with reporter type identified as a key feature. **Conclusions**: RF model demonstrated optimal predictive performance, with reporter type as the most important feature for detecting serious cases. This study emphasizes the importance of incorporating patient-reported data and polypharmacy surveillance to facilitate early detection of serious cases.

## 1. Introduction

Substance use disorder (SUD) has emerged as a major global public health challenge, characterized by compulsive substance use despite harmful physical or psychological effects, often leading to clinically significant functional impairment [1]. According to the Diagnostic and Statistical Manual of Mental Disorders (DSM)-5, SUD encompasses maladaptive behaviors involving alcohol, illicit drugs, and both prescription and non-prescription medications [2]. Although the term SUD encompasses a broad spectrum of substance-related pathologies, prescription drug use disorder (DUD) is restricted to the clinical diagnosis of problematic prescription drug use [3]. Notably, a recent study demonstrated that mortality associated with DUD increased from 1.83 deaths per 1,000,000 population in 1990 to 13.09 in 2021 and is projected to nearly double to 25.05 by 2040 [3]. Accordingly, it is imperative to differentiate this formal diagnostic entity from associated behavioral and physiological phenomena: ‘misuse’, defined as the use of medication outside of prescribed parameters, ‘abuse’ which refers to the intentional and non-therapeutic use of a substance to achieve a desired psychological effect, and ‘dependence’, which primarily manifests as a neurobiological or physiological adaptation resulting from chronic exposure [4].

The inappropriate use of medications with high dependence potential, including opioids, stimulants, sedatives, anxiolytics, and hypnotics, represents a major risk factor for DUD and often involves patterns of non-medical use or medication misuse [5,6,7,8]. Although these medications are primarily prescribed under medical supervision, they may also be accessed through non-medical or illicit channels, which can influence patterns of misuse, abuse, and dependence observed in real-world settings [5,6,7,8]. Such patterns not only contribute to the development of dependence but also elevate the risk of adverse drug events (ADEs), such as overdose, withdrawal symptom, and drug-related toxicity [5,6,7,8]. These misuse-related ADEs account for a substantial portion of healthcare utilization and emergency department visits, thereby exacerbating preventable morbidity and the overall public health burden [5,6,7,8,9]. Despite these concerns, early identification of patients at risk of serious abuse-, misuse-, and dependence-related outcomes remains limited in real-world clinical settings. Several studies have identified significant gaps in understanding the biological mechanisms and clinical predictors that contribute to the transition from therapeutic medication use to problematic use and dependence [10]. Moreover, a comprehensive evaluation of medication-specific risks is warranted, as dependence-prone medications comprise diverse pharmacological classes with distinct mechanisms of action [11,12]. Previous pharmacovigilance studies have primarily focused on either general adverse events or specific drug classes, with limited attention to misuse- and dependence-related ADEs in conjunction with their clinical severity.

Importantly, pharmacovigilance reporting systems offer a unique opportunity to capture real-world signals of medication abuse, misuse, and dependence, facilitating the identification of high-risk medications and vulnerable patient populations [11]. Pharmacovigilance databases can also capture early safety signals, as reports often include detailed clinical descriptions of medication abuse, misuse, withdrawal, or dependence-related events that may not be systematically recorded in other real-world data sources, such as electronic health records. Furthermore, reports may be submitted by either healthcare professionals or the general public; thus, differences in reporting behavior and clinical interpretation between these groups may influence the detection of safety signals and the identification of serious cases. However, the large volume and complexity of pharmacovigilance reports, which are often characterized by heterogeneous clinical information and non-linear relationships among patient- and drug-related factors, pose significant challenges for conventional analytical approaches [13]. Machine learning (ML) methods have emerged as powerful tools for analyzing high-dimensional real-world datasets and can facilitate the identification of complex patterns associated with ADEs [14]. Although ML has been increasingly applied in pharmacovigilance research, its use in predicting serious cases of medication abuse, misuse, and dependence remains limited. Integrating ML methodologies with pharmacovigilance data may therefore enhance the detection of clinically meaningful safety signals and improve risk stratification. Therefore, this study aimed to evaluate the reporting frequency of abuse-, misuse-, and dependence-related ADEs, as well as serious adverse events (SAEs), associated with medications with high dependence potential, to identify predictors associated with these outcomes, and to develop and validate ML models to predict serious cases of medication abuse, misuse, and dependence using nationwide pharmacovigilance data.

## 2. Materials and Methods

### 2.1. Database, Definition, and Data Acquisition

This study was conducted in accordance with the Strengthening the Reporting of Observational Studies in Epidemiology (STROBE) guidelines [15]. This study evaluated ADE cases reported to the Korean Adverse Event Reporting System Database (KIDS KAERS DB), a nationwide spontaneous voluntary reporting database constructed by the Korean Institute of Drug Safety and Risk Management (KIDS, Ministry of Food and Drug Safety), from January 2013 to December 2022 [16]. The study period was determined based on data availability at the time of extraction in 2023 to ensure a sufficiently long observation period for robust analysis while capturing temporal trends in abuse-, misuse- and dependence-related ADEs. In this study, medications with high dependence potential were defined as prescription drugs classified as narcotics or psychotropic substances under the Korean Narcotics Control Act, indicating their substantial risk of clinically significant abuse, misuse, dependence, and related adverse outcomes [17]. Although not classified as a narcotic under the Korean Narcotics Control Act, tramadol was included in the study due to its opioid pharmacological properties and well-documented potential for abuse, misuse, and dependence [18]. The etiologic medications were classified into 4 classes, opioids, anxiolytics, anesthetics, and hypnotics, based on their primary therapeutic clinical use and Anatomical Therapeutic Chemical (ATC) classification [10]. All ADE reports related to these medications were initially considered for inclusion. Inclusion criteria were ADE cases reported with “certain,” “probable/likely” and “possible” causality according to World Health Organization-Uppsala Monitoring Center (WHO-UMC) criteria [19]. Exclusion criteria included ADEs involving medications manufactured by fewer than two companies (MSK coded cases) to ensure data reliability. All cases were verified by KIDS-designated healthcare professionals through medical record review, patient interviews, and manufacturer pharmacovigilance data to minimize reporting bias [16]. All ADEs were coded according to the Medicinal Dictionary for Regulatory Activities (MedDRA) and further classified into system organ classes (SOC). SAEs were defined per International Conference on Harmonization (ICH) E2D guidelines, encompassing death, hospitalization, prolongation of existing hospitalizations, life-threatening ADEs, persistent or significant incapacity, or substantial disruption of the ability to conduct normal life functions, congenital abnormalities, birth defects, or other medically significant events [20]. Cases related to medication abuse, misuse, and dependence were defined as ADE reports coded under the MedDRA preferred terms “abuse”, “misuse”, and “dependence”.

The following information was extracted: (1) patient demographics, (2) medication lists, (3) ADE types, and (4) causality/severity assessments. When multiple events were reported for the same patient, each event was analyzed as an independent case, consistent with previous pharmacovigilance studies using spontaneous reporting system [21]. Access to the KIDS KAERS DB was granted by KIDS following formal approval procedures, and the use of anonymized data complied with relevant regulatory and ethical requirements. The study protocol was approved by the KIDS (KIDS KAERS DB 2308A0004) and the Institutional Review Board (IRB) of Kyung Hee University (KHSIRB-23-535).

### 2.2. Statistical Analysis

Patient demographics and ADE types were summarized using descriptive statistics. The Kolmogorov–Smirnov normality test was used to evaluate data normality, and age was presented as the median and interquartile range (IQR). Disproportionality analyses were conducted to detect association between medications and SAEs as well as between SOC-based ADEs and seriousness, and to determine the likelihood of reporting SAEs and ADEs related to abuse, misuse, and dependence for drugs with at least 4 reported cases, stratified by outcomes [21,22]. The effect size of the disproportionality analysis was estimated by reporting odds ratios (RORs), proportional reporting ratios (PRRs), and information components (ICs), along with corresponding 95% confidence intervals (CIs) and Mantel-Haenszel-adjusted *p*-values. Univariate logistic regression analysis was first performed to identify potential predictors of SAEs and ADEs related to abuse, misuse, and dependence. Subsequently, multivariate logistic regression with the forward selection method based on statistical significance was performed to estimate adjusted odds ratios (ORs) with 95% CI. All analyses were performed using R (version 4.3.3) and SPSS Statistics 26.0 (IBM SPSS Statistics for Windows, Armonk, NY, USA), with statistical significance set at *p*-values < 0.05.

### 2.3. Development and Validation of ML Models for Detecting Serious Abuse, Misuse, and Dependence Cases

A total of 16 predictors, which were further categorized into patient-related, drug-related, and report-related factors, were collected from the KIDS KAERS DB and included in the model. Patient-related variables included sex, age, and prior history of anxiety or insomnia. Drug-related variables included etiologic drugs, dosage route, number of concomitant drugs, and concomitant drug classes (non-steroidal anti-inflammatory drugs (NSAIDs), antidepressants, anticonvulsants, antipsychotics, acetaminophen and antidiabetic medications). Report-related variables included reporting year, reporter type, and sender of reports. The dependent variable was the occurrence of serious cases of abuse-, misuse-, and dependence-related ADEs. The dataset was randomly divided into model-building and model-testing datasets (70:30). Missing values were imputed using the most frequent value, and categorical variables were numerically encoded. Three ML algorithms were evaluated for predicting serious cases: random forest (RF), support vector machine (SVM), and eXtreme Gradient Boosting (XGBoost). Model training and hyperparameter optimization were performed using the training dataset with GridSearch. Class weighting was applied to mitigate class imbalance due to imbalance between serious and non-serious cases, and both feature selection and threshold tuning were conducted to optimize predictive performance. Model performance was evaluated on the test dataset using key metrics, including accuracy, sensitivity, specificity, balanced accuracy, kappa statistic, and area under the receiver operating characteristic curve (AUC-ROC). Optimal classification thresholds were determined using AUC-ROC curve analysis, and performance indicators were derived from the confusion matrix. To enhance model interpretability, the RF and XGBoost models were further analyzed using feature importance plots, local interpretable model-agnostic explanations (LIME), and permutation-based importance analysis. All analyses were conducted in R (version 4.3.3) using packages, including randomForest, xgboost, e1071, caret, pROC, lime, vip, and DALEX.

## 3. Results

### 3.1. Baseline Characteristics

**Figure 1:**
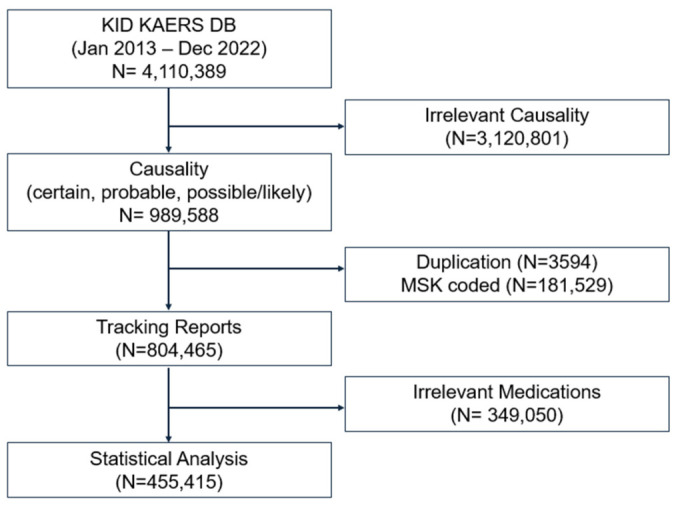
Flowchart of data acquisition. MSK-coded is designated for medications manufactured by fewer than two companies.

Among 4,110,389 ADE reports extracted from the KIDS KAERS DB, 455,415 reports involving medications with high dependence potential were included in the final analysis (Figure 1). Baseline characteristics are summarized in Table 1. The largest proportion of ADEs was reported in patients aged ≥ 60 years (n = 194,727; 42.76%), and 66.61% of reports involved women. Opioids were the most frequently reported suspected drug class (n = 427,005; 93.76%), with tramadol being the most commonly reported individual drug (n = 192,802; 42.34%). The overall occurrence of SAEs was 1.60% (n = 7429).

### 3.2. ADE Types and Risk of Reporting SAEs

**Figure 2:**
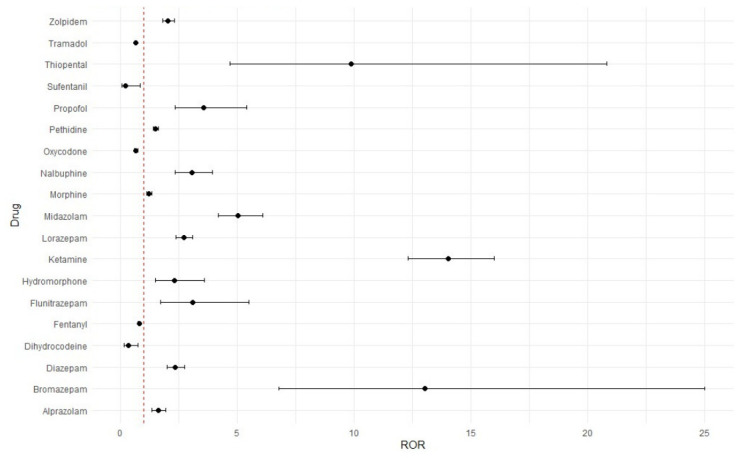
Association of SAEs with etiologic medications. The dotted red line indicates a ROR of 1.

Reports involving ketamine had the highest likelihood of being classified as SAEs (ROR 14.03, 95% CI 12.31–16.00), followed by bromazepam (ROR 13.02, 95% CI 6.70–25.00) and thiopental (ROR 9.86, 95% CI 4.67–20.82) (Figure 2). Only 5 agents, including dihydrocodeine (ROR 0.33, 95% CI 0.15–0.74), fentanyl (ROR 0.79, 95% CI 0.75–0.83), oxycodone (ROR 0.65, 95% CI 0.58–0.73), sufentanil (ROR 0.21, 95% CI 0.05–0.84) and tramadol (ROR 0.65, 95% CI 0.61–0.68) were associated with a significantly lower likelihood of SAEs. The highest reporting likelihood of SAEs was observed for cardiovascular disorders (ROR 30.36, 95% CI 28.58–32.25), followed by respiratory system disorders (ROR 17.03, 95% CI 15.93–18.20) and resistance mechanism disorders (ROR 8.04, 95% CI 2.93–22.84) (Table 2). The association between SOC classes and the seriousness of ADEs across medication classes is presented in Supplementary Tables (Tables S1–S4).

### 3.3. ADEs Related to Medication Abuse, Misuse, and Dependence

**Figure 3:**
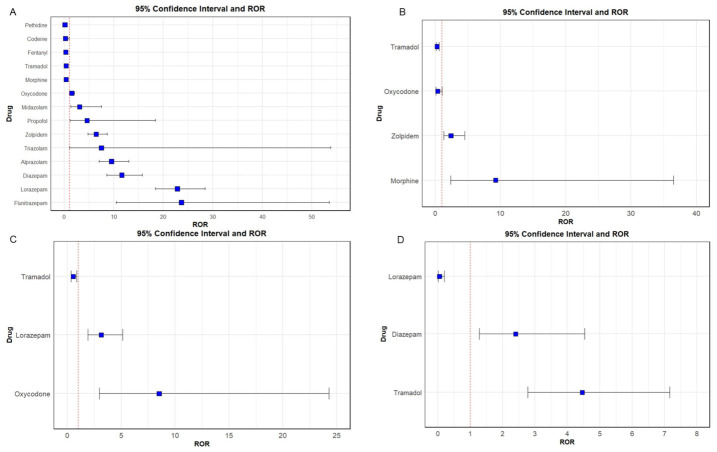
Disproportionality analysis of medications associated with abuse-, misuse-, and dependence-related ADEs. (**A**) overall cases, (**B**) medication abuse, (**C**) medication dependence and (**D**) medication misuse. The dotted red line indicates a ROR of 1.

The overall occurrence of medication abuse, misuse, and dependence was 0.11%. Significantly higher reporting likelihoods of abuse-, misuse-, and dependence-related ADEs were observed for several medications, including alprazolam (ROR 9.54, 95% CI 6.99–13.03), diazepam (ROR 11.63, 95% CI 8.57–15.79), flunitrazepam (ROR 23.68, 95% CI 10.48–53.50), lorazepam (ROR 22.86, 95% CI 18.39–28.43), midazolam (ROR 3.10, 95% CI 1.28–7.50), oxycodone (ROR 1.49, 95% CI 1.09–2.05), propofol (ROR 4.59, 95% CI 1.14–18.45), triazolam (ROR 7.50, 95% CI 1.05–53.80), and zolpidem (ROR 6.43, 95% CI 4.78–8.65) (Figure 3A). The detailed PRR and IC values associated with medication abuse, misuse, and dependence, stratified by etiologic medication, are summarized in Table S5. When stratified by outcome types, abuse was more frequently reported for morphine (ROR 9.26, 95% CI 2.35–36.48) and zolpidem (ROR 2.41, 95% CI 1.29–4.52) (Figure 3B). Dependence was more frequently reported for lorazepam (ROR 3.15, 95% CI 1.93–5.15) and oxycodone (ROR 8.52, 95% CI 2.99–24.27), whereas misuse-related ADEs were more frequently reported for diazepam (ROR 2.40, 95% CI 1.27–4.54) and tramadol (ROR 4.46, 95% CI 2.78–7.16) (Figure 3C,D). The detailed signal detections of medication abuse, misuse, and dependence are presented in Table 3.

### 3.4. Predictors Associated with SAEs and Medication Abuse, Misuse, and Dependence

The univariate analysis identified patient sex, age, number and type of concomitant medications, and drug classes as predictors associated with SAEs induced by medications with high dependence potential (Table 4). The multivariate analysis demonstrated a substantially decreased risk of SAEs in female sex (OR 0.65, 95% CI 0.62–0.68) and in patients aged <20 years (Table 4). The risk of SAEs was significantly increased with concomitant use of anticonvulsants (OR 1.45, 95% CI 1.23–1.69) and antipsychotics (OR 1.46, 95% CI 1.14–1.86). Meanwhile, concomitant administration of NSAIDs (OR 0.83, 95% CI 0.75–0.92), acetaminophen (OR 0.62, 95% CI 0.57–0.68) and antidepressants (OR 0.44, 95% CI 0.34–0.57) was associated with lower risk of SAEs. The risk of SAE was substantially higher with anxiolytics (OR 2.32, 95% CI 2.09–2.56), anesthetics (OR 4.24, 95% CI 3.73–4.82), and sedatives and hypnotics (OR 2.37, 95% CI 2.13–2.64) when compared to opioids.

The number and types of concomitant medications, ADE reporter type, and etiological medication classes were identified as major predictors of reports related to abuse, misuse, and dependence (Table 5). Multivariate logistic regression showed a significantly increased likelihood of these outcomes associated with concomitant use of acetaminophen (OR 3.60, 95% CI 2.40–5.39), antidepressants (OR 1.75, 95% CI 1.17–2.61), antipsychotics (OR 4.97, 95% CI 3.21–7.17), and anticonvulsants (OR 3.88, 95% CI 2.84–5.30) (Table 5). Compared with opioids, the likelihood was substantially higher for anxiolytics (OR 14.87, 95% CI 11.04–20.03) and sedatives and hypnotic agents (OR 7.81, 95% CI 5.36–11.38). Additionally, reports submitted by the general public were associated with a significantly higher likelihood of abuse-, misuse-, and dependence-related ADEs compared with those submitted by physicians (OR 4.59, 95% CI 3.04–6.94).

### 3.5. ML Models for Predicting Serious Medication Abuse, Misuse, and Dependence Cases

**Figure 4:**
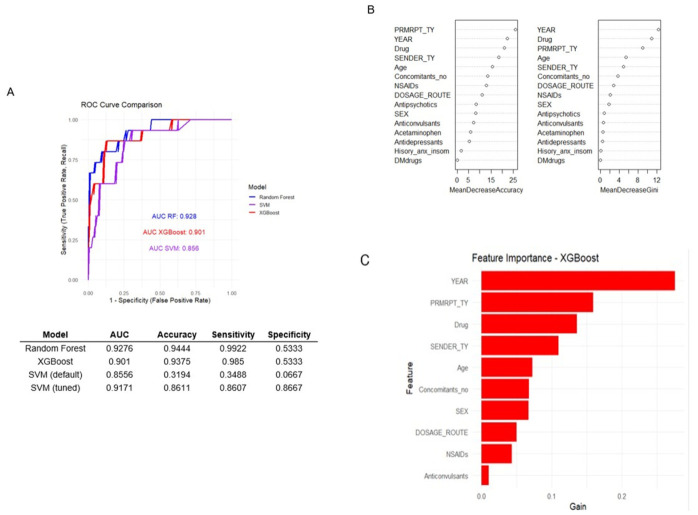
ML models for predicting serious abuse-, misuse-, and dependence-related ADEs. (**A**) comparison of machine learning models, (**B**) feature importance of RF, and (**C**) feature importance of XGBoost.

Three machine learning models, RF, XGBoost, and SVM, were developed to predict serious cases related to abuse, misuse, and dependence. Among the three models, RF model demonstrated the highest predictive performance for identifying serious cases (Figure 4). The RF model achieved an AUC–ROC of 0.928, with an overall accuracy of 94.4%, sensitivity of 99.2%, and specificity of 53.3%. Based on these performance metrics, the RF model was selected as the optimal model for further interpretation (κ = 0.6384; balanced accuracy = 0.76). The XGBoost model also demonstrated strong performance, with an AUC–ROC of 0.901, accuracy of 93.8%, and sensitivity of 98.4%, although its specificity remained similar (53.3%). The baseline SVM model showed relatively low performance (AUC = 0.856; accuracy = 31.9%). However, after variable selection and threshold tuning, its performance improved substantially (AUC = 0.917; accuracy = 86.1%). Feature importance analysis identified reporting year, reporter type, drug class, and patient age as key predictors in both the RF and XGBoost models.

## 4. Discussion

This study analyzed ADE reports related to medications with high dependence potential and identified predictors associated with SAEs and medication abuse, misuse, and dependence, including serious cases, based on pharmacovigilance investigations and ML-based prediction models. This study demonstrated a prevalence of 1.60% for SAEs and 0.11% for medication abuse, misuse, and dependence. Although the overall occurrence of these ADEs was relatively low, several medications, particularly benzodiazepines and opioids, showed a significantly higher reporting likelihood for medication abuse, misuse, and dependence. Concomitant use of central nervous system (CNS)-active medications, including antidepressants, antipsychotics, anticonvulsants, and acetaminophen, was associated with an increased risk of abuse-, misuse-, and dependence-related ADEs, implying an imperative role of polypharmacy. ML models demonstrated strong predictive performance in identifying serious cases, with the RF model achieving the highest performance. Notably, reporter type emerged as one of the most important features, with reports submitted by the general public exhibiting a higher likelihood of containing medication abuse, misuse, and dependence events compared to those from healthcare professionals, particularly physicians.

This study suggests that polypharmacy involving CNS-active agents may increase vulnerability to medication abuse, misuse, and dependence, particularly in patients with underlying psychiatric or neurological conditions. This finding is consistent with previous literature indicating that psychiatric comorbidities are associated with an increased risk of substance misuse [23]. Consistent with our findings, the Opioid Risk Tool (ORT), a validated instrument for predicting the risk of opioid misuse, identifies psychiatric disorders such as depression, schizophrenia, and bipolar disorder as major risk factors [24]. However, whether the elevated risk observed in our study is primarily driven by pharmacological effects or by underlying comorbidities remains unclear. Patients receiving antidepressants or antipsychotics may already have underlying psychiatric vulnerabilities that increase susceptibility to substance misuse, which may confound the observed associations. These findings imply the need for careful risk assessment when prescribing medications with high dependence potential in combination with CNS-active medications, particularly in patients with psychiatric comorbidities. The underlying mechanisms may involve dysregulation of dopaminergic reinforcement pathways, including chronic exposure to high-risk medications that trigger neuroadaptive changes, such as pharmacologic tolerance and the desensitization of the reward system [25]. Moreover, in patients with psychiatric comorbidities, this transition is often accelerated by a self-medication pathway, wherein individuals escalate dosages to alleviate un-resolved psychological distress or inadequately treated symptoms [26,27]. Further studies are needed to clarify these mechanisms and support safer use of medications with dependence potential. Particularly, prospective studies integrating clinical, behavioral and pharmacological data are warranted to demonstrate the relative contributions of drug-related effects and patient-level vulnerabilities.

Disproportionality analysis demonstrated a significantly higher reporting likelihood of misuse-related ADEs for diazepam and tramadol. The association observed with acetaminophen may partly reflect the frequent use of tramadol/acetaminophen combination products, potentially leading to unintentional exposure [28]. Moreover, in Korea, tramadol is not currently regulated as a narcotic analgesic under national drug control policies, and is therefore often perceived as a safer alternative to conventional opioids [29,30]. This regulatory status may facilitate broader prescribing and easier accessibility in routine clinical practice. Previous nationwide studies have also reported that tramadol is one of the most widely used analgesics in Korea, particularly among older adults, and that tramadol-related adverse events constitute a substantial proportion of analgesic-related safety reports [18,30,31]. These regulatory and prescribing contexts, combined with the perception of a relatively low misuse risk, may increase inappropriate or prolonged use in clinical settings, requiring cautious interpretation. Therefore, tramadol should be prescribed with caution, and strengthened clinical monitoring may be warranted to ensure its safe use.

One notable finding of this study was the significant role of reporter type in identifying serious cases related to abuse, misuse, and dependence. Feature importance analysis indicated that reporter type was one of the strongest predictors of serious cases, and logistic regression showed a substantially higher likelihood of these events being reported by the general public than by healthcare professionals. This discrepancy may reflect differences in the recognition and perception of medication abuse, misuse, and dependence between patients and healthcare professionals [32]. Patient reports may capture real-world experiences and symptoms that are not fully recognized in clinician-based reporting systems, whereas healthcare professionals may interpret these events more conservatively within a clinical context [32,33]. These findings accentuate the importance of public engagement in pharmacovigilance and the value of patient-reported safety information in strengthening drug safety surveillance. Moreover, pharmacists may also play a pivotal role in preventing medication abuse, misuse, and dependence through medication review, patient counseling, and early identification of high-risk individuals. Their involvement in multidisciplinary care has been shown to improve medication safety and optimize therapeutic outcomes.

Although patients are often assumed to have limited awareness of medication-related ADEs, no objective laboratory tests exist to detect medication abuse, misuse, and dependence, making patient reports an essential source for early signal detection [2,34]. Therefore, proactive patient participation and improved patient education may facilitate the earlier identification of maladaptive medication use [34,35]. Furthermore, the integration of patient-reported outcome (PRO) tools may enable systematic capture of patient experiences and support timely intervention [36]. Additionally, implementation of targeted warning systems, such as clinical decision-support tools based on pharmacovigilance data or ML models, may further enhance early detection of high-risk cases. To support practical implementation, pilot studies or questionnaire-based surveys among healthcare providers are needed to assess the feasibility, clinical acceptance, and utility of such systems. These approaches may ultimately contribute to reducing the risk of medication abuse, misuse, and dependence in routine clinical settings.

In addition to reporter type, reporting year was also identified as an important predictor in ML models. ADEs related to abuse, misuse, and dependence peaked during 2017–2020, which may reflect increased awareness driven by national campaigns, media attention, and evolving regulations [37]. Following the issuance of safe-use guidelines for controlled substances by the Ministry of Food and Drug Safety in 2021, reporting patterns appeared to change. However, this period also coincided with the COVID-19 pandemic, which altered healthcare utilization and prescribing patterns [38]. Therefore, further studies are warranted to disentangle the independent effects of regulatory interventions and pandemic-related societal changes on medication abuse, misuse, and dependence.

From a regulatory perspective, these findings imply the importance of strengthening drug safety surveillance systems through the integration of pharmacovigilance data with advanced analytical approaches. Future efforts may include collaboration with national health authorities to develop standardized regulatory systems, integrate pharmacovigilance data into real-time monitoring systems, and develop coordinated risk management strategies for high-risk medications. Such initiatives could enhance early signal detection and reduce the burden of misuse, dependence, and associated adverse outcomes at the population level. The identification of patient-reported factors, particularly reports from the general public, suggests the value of incorporating structured patient-reported information into pharmacovigilance systems to improve early signal detection for medication abuse, misuse, and dependence. In addition, the observed impact of polypharmacy emphasizes the need for regulatory strategies that account for concomitant drug use when evaluating drug safety in patients taking medications with high dependence potential. Furthermore, the application of machine learning models in this study demonstrates their potential to support proactive risk identification and inform regulatory decision-making, ultimately enhancing drug safety and public health protection.

To our knowledge, this is one of the first nationwide pharmacovigilance studies integrating machine learning to predict serious medication abuse, misuse, and dependence. The strengths of this study include the identification of key predictors of serious abuse-, misuse-, and dependence-related ADEs by integrating large-scale real-world pharmacovigilance data with ML methodologies. These findings may inform regulatory, clinical, and public health strategies to improve surveillance and prevent medication abuse, misuse, and dependence. However, several limitations should be considered when interpreting the findings. First, spontaneous reporting systems may contain incomplete or missing information, and underreporting of important clinical characteristics such as comorbidities may introduce bias and limit the ability to fully characterize patient populations. Additionally, because these systems lack a structured denominator for drug exposure, the findings should be interpreted as pharmacovigilance signals rather than direct measures of risk. Causal inference is also limited by the observational design. Second, although the random forest model demonstrated high sensitivity (99.2%) for detecting serious cases, its relatively low specificity (53.3%) suggests a potential for false-positive predictions. While high sensitivity supports signal detection, further refinement and external validation are required before clinical implementation. Third, the observed associations between psychotropic medications and misuse-related ADEs may partly reflect confounding by indication, as psychiatric disorders themselves are well-established risk factors for substance use disorders. Finally, the observed occurrence of misuse-related ADEs (0.11%) should not be interpreted as population-level prevalence because spontaneous reporting systems are subject to substantial underreporting and selective reporting. Behavioral outcomes such as abuse, misuse, and dependence are particularly prone to underrecognition and underreporting, potentially leading to underestimation of their true clinical burden. Despite these limitations, this study provides novel real-world evidence on risk patterns associated with serious medication abuse, misuse, and dependence and implies the potential role of ML approaches in improving medication safety surveillance. Further studies incorporating external validation and prospective data collection are warranted to enhance clinical applicability.

## 5. Conclusions

This study demonstrated that the integration of pharmacovigilance data with machine learning can effectively identify high-risk patterns of serious medication abuse, misuse, and dependence. Concomitant use of acetaminophen, antidepressants, antipsychotics, and anticonvulsants was identified as an important predictor associated with abuse-, misuse-, and dependence-related ADEs. In addition, reporter type, particularly reports from the general public, was also strongly associated with medication abuse, misuse, and dependence. These findings imply the value of pharmacovigilance-based surveillance in identifying high-risk medication use patterns and emphasize the importance of integrating patient-reported information into drug safety monitoring systems for early detection of medication abuse, misuse, and dependence. Strengthening pharmacovigilance and developing structured PRO-based monitoring tools may facilitate earlier detection and prevention of medication abuse, misuse, and dependence.

## Figures and Tables

**Table 1:** Demographic information.

Characteristics	No. of Cases (% Relative Frequency)
**Sex ^a^**	
Men	147,254 (32.33%)
Women	303,344 (66.61%)
**Age ^b^ Median (IQR)**	57 (23)
<20	16,685 (3.66%)
20~39	78,333 (17.20%)
40~59	159,359 (34.99%)
60~79	168,070 (36.90%)
≥80	26,657 (5.85%)
**Causality**	
Certain	7680 (1.69%)
Probable/Likely	153,350 (33.67%)
Possible	294,385 (64.64%)
**Seriousness**	
Non-serious ADEs	447,986 (98.37%)
Serious ADEs	7429 (1.63%)
**No. of Concomitantly Used Medications**	
1	299,401 (65.74%)
2	87,087 (19.12%)
3	26,672 (5.86%)
4	14,442 (3.17%)
≥5	27,813 (6.11%)
**Etiologic Drug Classes**	
Opioids	427,005 (93.76%)
Anxiolytics	14,620 (3.21%)
Sedative & Hypnotics	9846 (2.16%)
Anesthetics	3944 (0.87%)
**Medications**	
Tramadol	192,802 (42.34%)
Fentanyl	125,852 (27.63%)
Pethidine	45,235 (9.93%)
Oxycodone	27,367 (6.01%)
Morphine	24,557 (5.39%)
Codeine	8246 (1.81%)
Zolpidem	7916 (1.74%)
Lorazepam	5580 (1.23%)
Alprazolam	4783 (1.05%)
Diazepam	4135 (0.91%)
Ketamine	1538 (0.34%)
Midazolam	1536 (0.34%)
Nalbuphine	1278 (0.28%)
Remifentanil	1273 (0.28%)
Dihydrocodeine	1098 (0.24%)
Sufentanil	579 (0.13%)
Hydromorphone	570 (0.13%)
Propofol	415 (0.09%)
Flunitrazepam	248 (0.05%)
Triazolam	127 (0.03%)
Alfentanil	82 (0.02%)
Bromazepam	62 (0.01%)
Thiopental	57 (0.01%)
Clotiazepam	32 (0.01%)
Clobazam	26 (0.00%)
Zopiclone	19 (0.00%)
Chlordiazepoxide	2 (0.00%)

^a^ missing in 4817 (1.06%) cases; ^b^ missing in 6311 (1.39%) cases.

**Table 2:** System Organ Class (SOC)-based ADEs.

System Organ Class	*p*-Value	ROR (95%CI)	PRR (95% CI)	IC (IC025–0975)
Body as a whole-general disorders	<0.001	3.26 (3.02–3.52)	3.15 (2.93–3.39)	1.55 (1.44–1.66)
Cardiovascular disorders, general	<0.001	30.36 (28.58–32.25)	22.19 (21.17–23.26)	4.09 (4.00–4.17)
Central & peripheral nervous system disorders	<0.001	0.86 (0.81–0.91)	0.86 (0.81–0.91)	−0.18 (−0.26, −0.09)
Gastro-intestinal system disorders	<0.001	0.07 (0.07–0.08)	0.08 (0.07–0.08)	−2.63 (−2.74, −2.52)
Hearing and vestibular disorders	0.043	2.32 (1.03–5.25)	2.27 (1.04–4.98)	1.05 (−0.08–2.18)
Heart rate and rhythm disorders	<0.001	6.04 (5.30–6.88)	5.59 (4.97–6.30)	2.43 (2.24–2.62)
Liver and biliary system disorders	<0.001	4.08 (3.44–4.85)	3.89 (3.31–4.57)	1.93 (1.68–2.17)
Metabolic and nutritional disorders	0.097	1.39 (0.94–2.05)	1.38 (0.95–2.02)	0.45 (−0.1–1.01)
Musculo-skeletal system disorders	<0.001	2.21 (1.51–3.25)	2.17 (1.50–3.15)	1.09 (0.54–1.64)
Platelet, bleeding & clotting disorders	<0.001	5.81 (3.03–11.11)	5.38 (2.98–9.74)	2.15 (1.24–3.07)
Psychiatric disorders	0.289	1.05 (0.96–1.16)	1.05 (0.96–1.16)	0.07 (−0.07–0.21)
Resistance mechanism disorders	<0.001	8.04 (2.93–22.84)	7.22 (2.87–18.12)	2.09 (0.68–3.51)
Respiratory system disorders	<0.001	17.03 (15.93–18.20)	13.95 (13.2–14.75)	3.55 (3.46–3.64)
Secondary terms-events	0.028	2.50 (1.10–5.65)	2.44 (1.11–5.34)	1.13 (0, 2.26)
Skin and appendages disorders	0.558	1.03 (0.94–1.12)	1.03 (0.94–1.12)	0.035 (−0.09–0.16)
Urinary system disorders	0.307	1.11 (0.91–1.35)	1.11 (0.91–1.34)	0.14 (−0.14–0.43)
Vascular (extracardiac) disorders	<0.001	3.70 (2.49–5.50)	3.54 (2.44–5.15)	1.75 (1.19–2.32)
Vision disorders	<0.001	2.78 (1.90–4.06)	2.70 (1.88–3.89)	1.39 (0.84–1.93)
White cell and RES * disorders	0.047	1.76 (1.01–3.04)	1.73 (1.01–2.96)	0.75 (−0.03–1.53)

* RES: reticuloendothelial system.

**Table 3:** Signal detection of medication abuse, misuse, and dependence.

Medication	ROR (95% CI)	PRR (95% CI)	IC (IC025–IC975)
**Abuse**			
Morphine	9.26 (2.35–36.48)	4.39 (2.61–7.36)	1.67 (0.29–3.05)
Zolpidem	2.41 (1.29–4.52)	2.85 (1.64–4.93)	0.94 (0.12–1.77)
Oxycodone	0.37 (0.13–1.05)	0.45 (0.17–1.21)	−0.89 (−2.32–0.54)
Tramadol	0.29 (0.14–0.61)	0.30 (0.15–0.61)	−1.05 (−2.05, −0.05)
**Dependence**			
Oxycodone	8.52 (2.99–24.27)	1.49 (1.29–1.73)	0.46 (−0.13–1.05)
Lorazepam	3.15 (1.93–5.15)	1.32 (1.10–1.57)	0.21 (−0.14–0.57)
Tramadol	0.56 (0.36–0.87)	0.56 (0.44–0.70)	−0.55 (−1.00, −0.10)
**Misuse**			
Tramadol	4.46 (2.78–7.16)	3.52 (2.23–5.56)	0.79 (0.28, 1.30)
Diazepam	2.40 (1.28–4.54)	1.63 (1.06–2.51)	0.53 (−0.26, 1.32)
Lorazepam	0.05 (0.01–0.20)	0.04 (0.01–0.17)	−3.52 (−5.33, −1.70)

**Table 4:** Predictors associated with serious adverse events.

Predictors	Overall Classes	Opioids	Anxiolytics	Anesthetics	Sedatives & Hypnotics
**Sex**					
Men	1 (reference)				
Women	0.65 (0.62–0.68)	0.62 (0.58–0.65)	N/A	0.67 (0.51–0.87)	N/A
**Age**					
<20	1 (reference)				
20–39	0.48 (0.43–0.53)	0.77 (0.66–0.89)	0.65 (0.44–0.96)	0.08 (0.04–0.16)	0.18 (0.11–0.29)
40–59	0.55 (0.50–0.61)	0.94 (0.82–1.07)	0.42 (0.29–0.60)	0.18 (0.12–0.28)	0.16 (0.11–0.24)
60–79	0.64 (0.58–0.71)	1.09 (0.95–1.24)	0.41 (0.29–0.59)	0.23 (0.15–0.35)	0.23(0.16–0.33)
≥80	0.77 (0.68–0.87)	1.28 (1.09–1.51)	0.78 (0.51–1.20)	0.06 (0.01–0.44)	0.28 (0.18–0.44)
**No. Concomitantly Used Medications**					
1	1 (reference)				
2	1.12 (1.04–1.21)	1.01 (0.93–1.10)	1.79 (1.42–2.26)	0.88 (0.64–1.19)	2.21 (1.67–2.91)
3	1.71 (1.54–1.89)	1.83 (1.65–2.04)	0.88 (0.65–1.19)	1.50 (0.91–2.47)	1.55 (1.03–2.33)
4	1.49 (1.29–1.72)	1.60 (1.37–1.87)	0.90 (0.64–1.26)	1.90 (0.90–4.00)	1.01 (0.57–1.76)
≥5	1.00 (0.86–1.15)	1.41 (1.23–1.62)	0.25 (0.17–0.36)	N/A	0.29 (0.16–0.50)
**Types of Concomitantly Used Medications**					
NSAIDs	0.83 (0.75–0.92)		N/A	0.08 (0.02–0.34)	2.20 (1.12–4.30)
Acetaminophen	0.62 (0.57–0.68)	0.58 (0.53–0.63)	N/A	N/A	N/A
Anticonvulsants	1.45 (1.23–1.69)		2.94 (2.22–3.89)	N/A	N/A
Antidepressants	0.44 (0.34–0.57)	0.32 (0.14–0.71)	0.52 (0.38–0.73)	N/A	N/A
Antipsychotics	1.46 (1.14–1.86)		N/A	N/A	2.92 (1.72–4.96)
**Medication Classes**					
Opioids	1 (reference)	N/A			
Anxiolytics	2.32 (2.09–2.56)				
Anesthetics	4.24 (3.73–4.82)				
Sedatives & Hypnotics	2.37 (2.13–2.64)				

**Table 5:** Predictors of medication abuse-, misuse-, and dependence-related ADEs.

Predictors	Overall Classes	Opioids	Anxiolytics	Anesthetics	Sedatives & Hypnotics
**Sex**					
Men	1 (reference)				
Women	N/A	0.66 (0.48–0.92)	N/A	N/A	N/A
**Age**					
<20	N/A				
20–39					
40–59					
60–79					
≥80					
**No. Concomitantly Used Medications**					
1	1 (reference)				
2	0.39 (0.26–0.58)	0.44 (0.18–1.05)	0.26 (0.13–0.52)	N/A	N/A
3	0.25 (0.16–0.42)	0.26 (0.09–0.71)	0.26 (0.13–0.53)		
4	0.14 (0.07–0.25)	0.19 (0.07–0.57)	0.05 (0.01–0.22)		
**≥5**	0.17 (0.10–0.29)	0.12 (0.04–0.33)	0.33 (0.19–0.59)		
**Types of Concomitantly Used Medications**					
NSAIDs	N/A	N/A	N/A	N/A	5.59 (2.08–15.06)
Acetaminophen	3.60 (2.40–5.39)	4.42 (1.86–10.48)	N/A	N/A	0.10 (0.01–0.82)
Antidepressants	1.75 (1.17–2.61)	N/A	N/A	N/A	N/A
Antipsychotics	4.97 (3.21–7.71)	7.06 (0.89–56.07)	4.04 (2.43–6.72)	N/A	2.53 (1.06–6.05)
Anticonvulsants	3.42 (2.42–4.81)	3.48 (2.01–6.00)	2.91 (1.75–4.83)	N/A	N/A
**Reporting Personnel**					
Doctors	1 (reference)				
Pharmacists	1.18 (0.88–1.57)	3.64 (1.99–6.67)	0.65 (0.42–1.02)	N/A	0.37 (0.16–0.87)
Other Healthcare Professional	0.11 (0.08–0.16)	0.17 (0.09–0.33)	0.18 (0.10–0.32)		0.12 (0.05–0.30)
General Public	4.59 (3.04–6.94)	8.35 (4.01–17.36)	3.17 (1.51–6.67)		5.89 (2.44–14.21)
**Medication Classes**					
Opioids	1 (reference)	N/A			
Anxiolytics	14.87 (11.04–20.03)				
Anesthetics	1.41 (0.20–10.16)				
Sedatives & Hypnotics	7.81 (5.36–11.38)				

## Data Availability

The data presented in this study are available upon request from the corresponding author and KIDS owing to the inclusion of patient information and ethical concerns.

## Supplementary Materials

The following supporting information can be downloaded at: https://www.mdpi.com/article/10.3390/healthcare14101265/s1, Table S1: System Organ Class (SOC)-based ADEs caused by opioids; Table S2: System Organ Class (SOC)-based ADEs caused by anxiolytics; Table S3: System Organ Class (SOC)-based ADEs caused by anesthetics; Table S4: System Organ Class (SOC)-based ADEs caused by sedatives and hypnotics; Table S5: Association of medications with ADEs related to medication abuse, misuse, and dependence.

## Abbreviations

The following abbreviations are used in this manuscript:

ADE	Adverse Drug Events
CI	Confidence Interval
DUD	Drug Use Disorder
FDA	Food and Drug Administration
OR	Odds Ratio
RF	Random Forest
ROR	Reporting Odds Ratio
SVM	Support Vector Machine
SUD	Substance Use Disorder
XGBoost	eXtreme Gradient Boosting
