# Supplementary material for: Predicting Serious Adverse Events, Medication Abuse, Misuse, and Risk of Dependence for Medications with High Dependence Potential: Role of Patient-Reported Factors and Machine Learning Approach
Source: Healthcare (Basel). 2026 May 7;14(10):1265. doi: 10.3390/healthcare14101265 (PMC13205485; doi:10.3390/healthcare14101265)
Supplement: Supplementary file 1 [file healthcare-14-01265-s001.zip › healthcare-4293184-supplementary.pdf]

Table S1. System Organ Class (SOC)-based ADEs caused by opioids

System organ class	P-value	ROR (95%CI)	**PRR (95% CI)**	**IC (IC025-IC975)**
Body as a whole - general disorders	<0.001	3.63 (3.34-3.95)	3.51 (3.24-3.80)	1.70 (1.58-1.81)
Cardiovascular disorders, general	<0.001	36.89 (34.61-39.33)	26.63 (25.34-27.99)	4.29 (4.21-4.38)
Central & peripheral nervous system disorders	<0.001	0.84 (0.79-0.90)	0.84 (0.79-0.90)	-0.20 (-0.30, -0.11)
Gastro-intestinal system disorders	<0.001	0.07 (0.07-0.08)	0.08 (0.07-0.08)	-2.53 (-2.64, -2.41)
Hearing and vestibular disorders	0.008	3.00 (1.32-6.80)	2.92 (1.33-6.38)	1.35 (0.21-2.48)
Heart rate and rhythm disorders	<0.001	6.46 (5.62-7.43)	6.00 (5.28-6.82)	1.35 (0.21-2.48)
Liver and biliary system disorders	<0.001	4.06 (3.37-4.89)	3.89 (3.26-4.64)	1.92 (1.66-2.19)
Metabolic and nutritional disorders	0.82	1.06 (0.63-1.80)	1.06 (0.63-1.79)	0.08 (-0.67-0.83)
Musculoskeletal system disorders	0.163	1.48 (0.85-2.56)	1.47 (0.86-2.52)	0.53 (-0.67, 0.88)
Platelet, bleeding & clotting disorders	0.003	3.91 (1.59-9.63)	3.75 (1.60-8.80)	1.59 (0.34-2.83)
Psychiatric disorders	00.39	1.06 (0.93-1.21)	1.06 (0.93-1.20)	0.08 (-0.11-0.27)
Respiratory system disorders	<0.001	16.09 (14.90-17.36)	13.53 (12.68-14.44)	3.54 (3.43-3.65)
Skin and appendages disorders	0.077	1.09 (0.99-1.20)	1.09 (0.99-1.20)	0.11 (-0.02-0.25)
Urinary system disorders	0.009	1.31 (1.07-1.59)	1.30 (1.07-1.58)	0.37 (0.08-0.66)
Vascular (extracardiac) disorders	<0.001	4.11 (2.72-6.21)	3.93 (2.67-5.81)	1.89 (1.30-2.48)
Vision disorders	<0.001	2.76 (1.70-4.49)	2.69 (1.69-4.30)	1.36 (0.67-2.05)
White cell and RES* disorders	0.179	1.57 (0.81-3.05)	1.56 (0.82-2.98)	0.60 (-0.33-1.53)

Table S2. System Organ Class (SOC)-based ADEs caused by anxiolytics

System organ class	P-value	ROR (95%CI)	PRR (95% CI)	IC (IC025-IC975)
Body as a whole - general disorders	<0.001	1.82 (1.39-2.37)	1.77 (1.38-2.27)	0.74 (0.36-1.11)
Cardiovascular disorders, general	<0.001	5.32 (3.98-7.11)	4.67 (3.64-5.99)	2.05 (1.64-2.46)
Central & peripheral nervous system disorders	0.382	1.10 (0.89-1.36)	1.10 (0.89-1.34)	0.10 (-0.19-0.40)
Gastro-intestinal system disorders	<0.001	0.20 (0.13-0.30)	0.21 (0.14-0.31)	-2.03 (-2.63, -1.43)
Heart rate and rhythm disorders	0.002	2.51 (1.41-4.45)	2.38 (1.40-4.03)	1.16 (0.35-1.97)
Liver and biliary system disorders	<0.001	8.28 (4.78-14.33)	6.60 (4.31-10.11)	2.48 (1.72-3.24)
Metabolic and nutritional disorders	0.838	0.92 (0.41-2.08)	0.92 (0.42-2.03)	-0.11 (-1.24-1.03)
Musculoskeletal system disorders	<0.001	3.63 (1.92-6.85)	3.32 (1.89-5.82)	1.57 (0.68-2.47)
Platelet, bleeding & clotting disorders	<0.001	13.60 (4.63-39.92)	9.40 (4.57-19.32)	2.41 (1.00-3.82)
Psychiatric disorders	<0.001	0.29 (0.22-0.37)	0.30 (0.23-0.38)	-1.34 (-1.69, -0.98)
Respiratory system disorders	<0.001	11.82 (8.96-15.59)	8.85 (7.17-10.92)	2.88 (2.50-3.26)
Skin and appendages disorders	0.137	1.28 (0.93-1.76)	1.27(0.93-1.72)	0.31 (-0.15-0.77)
Vision disorders	0.64	1.22 (0.54-2.78)	1.21 (0.55-2.66)	0.25 (-0.89-1.39)

Table S3. System Organ Class (SOC)-based ADEs caused by anesthetics

System organ class	P-value	ROR (95%CI)	PRR (95% CI)	IC (IC025-IC975)
Body as a whole - general disorders	0.135	1.47 (0.89-2.43)	1.42 (0.91-2.21)	0.46 (-0.25-1.17)
Cardiovascular disorders, general	<0.001	6.71 (4.41-10.20)	4.68 (3.53-6.20)	2.03 (1.48-2.59)
Central & peripheral nervous system disorders	0.055	0.70 (0.49-1.01)	0.72 (0.52-1.01)	-0.41 (-0.91-0.10)
Gastro-intestinal system disorders	<0.001	0.02 (0.01-0.04)	0.02 (0.01-0.05)	-4.22 (-5.15, -3.30)
Heart rate and rhythm disorders	<0.001	5.23 (2.86-9.56)	3.90 (2.57-5.94)	1.80 (0.98-2.61)
Psychiatric disorders	0.524	0.76 (0.33-1.76)	0.78 (0.36-1.70)	-0.33 (-1.49 – 0.82)
Respiratory system disorders	<0.001	20.18 (15.63-26.06)	11.68 (9.59-14.21)	2.40 (2.12-2.67)
Skin and appendages disorders	<0.01	0.33 (0.17-0.62)	0.35 (0.19-0.64)	-1.41 (-2.30,-0.51)

Table S4. System Organ Class (SOC)-based ADEs caused by sedatives and hypnotics

System organ class	P-value	ROR (95%CI)	PRR (95% CI)	IC (IC025-IC975)
Body as a whole - general disorders	0.236	0.78 (0.52-1.17)	0.79 (0.53-1.17)	-0.31 (-0.89-0.27)
Cardiovascular disorders, general	<0.001	9.06 (6.08-13.49)	7.02 (5.18-9.51)	2.59 (2.04-3.14)
Central & peripheral nervous system disorders	0.279	1.15 (0.89-1.48)	1.14 (0.90-1.45)	0.15 (-0.20-0.50)
Gastro-intestinal system disorders	<0.001	0.25 (0.14-0.42)	0.25 (0.15-0.43)	-1.80 (-2.55, -1.04)
Heart rate and rhythm disorders	0.018	2.59 (1.18-5.68)	2.44 (1.20-4.97)	1.14 (0.05-2.23)
Liver and biliary system disorders	<0.001	9.24 (2.65-25.68)	6.43 (2.74-15.12)	2.00 (0.49-3.51)
Metabolic and nutritional disorders	0.027	2.61 (1.12-6.08)	2.45 (1.14-5.28)	1.13 (-0.04-2.30)
Psychiatric disorders	<0.001	0.51 (0.41-0.64)	0.52 (0.42-0.65)	-0.59 (-0.89, -0.29)
Respiratory system disorders	<0.001	5.59 (4.28-7.30)	4.86 (3.85-6.12)	2.00 (1.63-2.37)
Secondary terms - events	0.012	4.93 (1.42-17.11)	4.28 (1.52-12.07)	1.54 (-0.12, 3.19)
Skin and appendages disorders	0.012	0.40 (0.20-0.82)	0.41 (0.21-0.83)	-1.18 (-2.17,-0.20)
Vision disorders	0.194	1.97 (0.71-5.49)	1.90 (0.74-4.90)	0.78 (-0.61-2.18)

Table S5. Association of medications with ADEs related to medication abuse, misuse, and dependence

Medications	p-values	ROR (95% CI)	PRR (95% CI)	IC (IC025-IC975)
Alprazolam	<0.001	9.54 (6.99-13.03)	9.47 (6.95-12.89)	3.00 (2.56-3.45)
Codeine	0.036	0.23 (0.06-0.91)	0.23 (0.06-0.91)	-1.88 (-3.68, -0.09)
Diazepam	<0.001	11.63 (8.57-15.79)	11.51 (8.51-15.58)	3.25 (2.82-3.69)
Fentanyl	<0.001	0.29 (0.22-0.39)	0.29 (0.22-0.39)	-1.46 (-1.89, -1.04)
Flunitrazepam	<0.001	23.68 (10.48-53.50)	23.14 (10.44-51.26)	3.09 (1.96-4.22)
Lorazepam	<0.001	22.86 (18.39-28.43)	22.45 (18.12-27.83)	4.04 (3.74-4.35)
Midazolam	0.012	3.10 (1.28-7.50)	3.10 (1.29-7.47)	1.38 (0.16-2.59)
Morphine	0.002	0.37 (0.20 – 0.70)	0.37 (0.20-0.70)	-1.34 (-2.22, -0.45)
Oxycodone	0.013	1.49 (1.09-2.05)	1.49 (1.09-2.05)	0.53 (0.08-0.98)
Pethidine	<0.001	0.13 (0.06-0.28)	0.13 (0.06-0.28)	-2.69 (-3.73, -1.65)
Propofol	0.032	4.59 (1.14-18.45)	4.57 (1.14-18.26)	1.41 (-0.39-3.21)
Tramadol	<0.001	0.37 (0.30-0.46)	0.37 (0.30-0.46)	-0.98 (-1.29, -0.68)
Triazolam	0.045	7.50 (1.05-53.80)	7.45 (1.06-52.61)	1.24 (-1.08, 3.57)
Zolpidem	<0.001	6.43 (4.78-8.65)	6.40 (4.77-8.59)	2.48 (2.06-2.90)
